# Can echocardiographic screening in the early days of life detect critical congenital heart disease among apparently healthy newborns?

**DOI:** 10.1186/s12887-018-1344-z

**Published:** 2018-11-19

**Authors:** Miyuki Kondo, Akira Ohishi, Toru Baba, Tomoka Fujita, Shigeo Iijima

**Affiliations:** Department of Pediatrics, Hamamatsu University School of Medicine, 1 – 20 – 1 Handayama, Hamamatsu, Shizuoka, 431 – 3192 Japan

**Keywords:** Critical congenital heart disease, Echocardiography, Screening, Newborn

## Abstract

**Background:**

Delayed diagnosis of critical congenital heart disease (CCHD) carries a serious risk of mortality, morbidity, and handicap. As echocardiography is commonly used to diagnose congenital heart disease (CHD), echocardiographic investigations in newborns may be helpful in detecting CCHD earlier and with higher sensitivity than when using other screening methods. The present study aimed to evaluate the effectiveness of echocardiographic screening for CCHD in a tertiary care center.

**Methods:**

A retrospective chart review was conducted among newborns delivered at Hamamatsu University Hospital between June 2009 and May 2016. The study included consecutive newborns who underwent early echocardiographic screening (within the first 5 days of life) performed by pediatric cardiologists, were born at ≥36 weeks of gestation, had a birthweight ≥2300 g, and were cared for in the well-baby nursery. Newborns admitted to the neonatal intensive care unit, as well as those with prenatal diagnosis of CHD and/or clinical symptoms or signs of CHD were excluded. Four CHD outcome categories were defined: critical, serious, clinically significant, and clinically non-significant.

**Results:**

A total of 4082 live newborns were delivered during the study period. Of 3434 newborns who met the inclusion criteria and had complete echocardiography data, 104 (3.0%) were diagnosed as having CHD. Among these, none was initially diagnosed as having critical or serious CHD. Of the 95 newborns who continued follow-up with a cardiologist, 61 (64%) were determined to have non-significant CHDs that resolved within 6 months of life. Review of excluded newborns revealed nine cases of critical or serious CHD; among these newborns, six were diagnosed prenatally and three had some clinical signs of CHD prior to hospital discharge.

**Conclusions:**

In our tertiary care center, echocardiography screening within the first 5 days of life did not help improve CCHD detection rate in newborns without prenatal diagnosis or clinical signs of CHD. Echocardiographic screening may be associated with increased rate of false-positives (defects resulting in clinically non-significant CHDs) in newborns without prenatal diagnosis or suspicion of CHD.

## Background

Heart defects are the most common congenital malformations, with an incidence of approximately eight per 1000 live births [1], while the incidence of critical congenital heart disease (CCHD) is one or two per 1000 newborn babies [2]. In general, CCHD is defined as congenital heart disease (CHD) that leads to death or requires surgery or catheter intervention within 28 days of life [3]. Delayed detection of CCHD increases the risk of acute cardiovascular collapse and death, and is associated with worse outcomes of interventions [2, 4]. As the preference for early discharge after delivery is becoming more prevalent, newborns with CCHD are more likely to develop symptoms at home rather than during their stay in the newborn nursery. Screening for CCHD has previously relied on prenatal ultrasound and postnatal clinical examination, but both such approaches are known to have a relatively low detection rate; it is estimated that up to a third of newborns may be discharged with undiagnosed critical defects [5]. For more than a decade, many studies have assessed the usefulness of pulse oximetry for improving the detection of CCHD in newborns before hospital discharge (typically, in the newborn nursery); evidence from such studies indicates that pulse oximetry screening is highly effective in detecting CCHD in newborns with hypoxemia [6, 7] but not in those without hypoxemia [3, 8]. Echocardiography, especially when performed by pediatric cardiologists, is commonly used for diagnosing CHD, and thus may be helpful in detecting CCHD in newborns earlier and with higher sensitivity than when using other screening methods. However, few studies have assessed the effectiveness of hospital-wide echocardiography screening for CHD in newborns [9–11]. The objective of the present study was to evaluate the effectiveness of echocardiographic screening for CCHD when evaluations are performed by pediatric cardiologists in a tertiary care center.

## Methods

### Study population

A retrospective chart review was conducted among newborns delivered at Hamamatsu University Hospital, Japan, to identify those who underwent echocardiographic screening between June 2009 and May 2016. Hamamatsu University Hospital is a tertiary care birthing center that provides extensive prenatal screening and serves as a referral hospital for pregnant women whose fetuses have been diagnosed with congenital anomalies at other medical facilities. In this hospital’s well-baby nursery, neonatologists conduct clinical examinations of newborns on the 1st and 4th day of life; echocardiographic screening is performed by a pediatric cardiologist in each live newborn, with written informed consent from the parents.

This study included consecutive newborns delivered at ≥36 weeks of gestation, having a birthweight ≥2300 g, and cared for in the well-baby nursery. Newborns admitted to the neonatal intensive care unit (NICU), as well as those prenatally diagnosed with CHD and/or with clinical signs or symptoms indicative of CHD before discharge were excluded. The study was approved by the ethics committee of our institution (Approval No: 17–277).

### Screening protocol

The newborns underwent echocardiographic screening within the first 5 days of life. The echocardiographic examination was performed by one of three well-experienced pediatric cardiologists, using the same echocardiography machine (Vivid i; GE Healthcare, Tokyo, Japan) with a 12-MHz transducer. The examination protocol included two-dimensional and color Doppler imaging in the parasternal, suprasternal, subxiphoid, apical, and, when necessary, modified views. All echocardiographic examinations were recorded and reviewed by another pediatric cardiologist, who served as a second observer blinded to the patient identity. Sedation was not required in any newborn because echocardiographic screening was performed during natural sleep. In newborns who had a ductal shunt at the time of the initial screening, echocardiography was performed again upon hospital discharge; the diagnosis of patent ductus arteriosus (PDA) was only made in newborns with a ductal shunt that persisted at the time of discharge. Atrial septal defect was defined as the presence of an intra-atrial communication ≥5 mm in diameter, with an enlarged right atrium and right ventricle. An intra-atrial defect ≤4 mm in size at the fossa ovalis was considered to represent patent foramen ovale. For the purpose of the present study, echocardiographic follow-up of patients with CHD was considered complete upon spontaneous resolution of all cardiac lesions, surgery, catheter intervention, or death. On the basis of the CHD severity classification proposed by Ewer and colleagues [3], four CHD outcome categories were defined in this study: (i) critical CHD, which was defined in infants with hypoplastic left heart syndrome, pulmonary atresia (PA) with intact ventricular septum, simple transposition of the great arteries, or interruption of the aortic arch (IAA), as well as in infants who died or required surgery within the first 28 days of life for coarctation of the aorta (CoA), aortic valve stenosis, pulmonary valve stenosis, tetralogy of Fallot (TOF), PA with ventricular septal defect, or total anomalous pulmonary venous connection (TAPVC); (ii) serious CHD, which was defined in infants with non-critical cardiac lesions requiring intervention (cardiac catheterization or surgery) within the first year of life; (iii) significant CHD, defined as any cardiac lesion persisting longer than 6 months of life but not classified as serious or critical; and (iv) non-significant CHD, defined as any defects not clinically appreciable (e.g., with spontaneous resolution) and not persisting after 6 months of life.

### Results

**Fig. 1:**
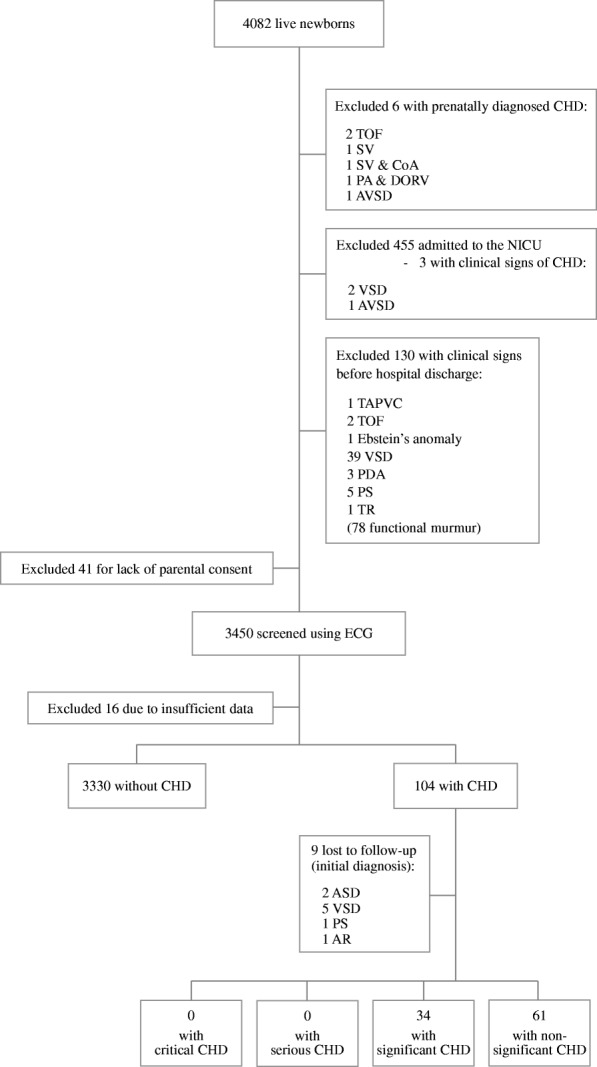
Study population and flow diagram showing the progress of newborns included in the study CHD, congenital heart disease; NICU, neonatal intensive care unit; TOF, tetralogy of Fallot; SV, single ventricle; CoA, coarctation of the aorta; PA, pulmonary atresia; DORV, double-outlet right ventricle; AVSD, atrial ventricular septal defect; TAPVC, total anomalous pulmonary venous connection; ASD, atrial septal defect; VSD, ventricular septal defect; PDA, patent ductus arteriosus; MR, mitral regurgitation; AR, aortic regurgitation; TR, tricuspid regurgitation; PS, pulmonary stenosis; ECG, echocardiography

During the study period, 4082 live newborns were delivered at ≥36 weeks of gestation and with a birthweight of ≥2300 g. The flow diagram showing the progress of newborns included in the present study is shown in Fig. 1. Newborns with prenatal diagnosis of CHD (*n* = 6), those admitted to the NICU (*n* = 455), and those who had signs or symptoms of CHD prior to hospital discharge (*n* = 130) were excluded. Of the remaining 3491 newborns, 41 were not screened due to lack of parental consent. Among the 3434 newborns with complete echocardiography data, 104 (3.0%) were diagnosed as having CHD, of whom nine were lost to follow-up because of dropping out or moving away. Of the remaining 95 newborns diagnosed as having CHD during the initial echocardiographic screening, 34 (36%) and 61 (64%) were determined to have significant and non-significant CHD, respectively; no cases of critical or serious CHD were noted. A summary of the types of cardiac lesions and the duration of follow-up for these 95 newborns with CHD is provided in Table 1. Of the 34 newborns diagnosed as having significant CHD, 18 (53%) experienced spontaneous resolution of cardiac lesions during the course of the 2-year follow-up period.

**Table 1:** Number of newborns with significant and non-significant congenital heart disease, stratified according to follow-up period

CHD	Follow-up period			
	< 1 month	1–6 months	6 months to 2 years	> 2 years
Significant				
ASD			3	3
VSD			12	2
PDA			1	4
MR			1	1
AR			1	3
PS				3
Non-significant				
VSD	2	11		
PDA	31	4		
MR	3	4		
AR	2	4		

*CHD* congenital heart disease, *ASD* atrial septal defect, *VSD* ventricular septal defect, *PDA* patent ductus arteriosus, *MR* mitral regurgitation, *AR* aortic regurgitation, *PS* pulmonary stenosis

Among the excluded newborns, nine (0.22% of all live births) were diagnosed as having critical (*n* = 2) or serious CHD (*n* = 7). The clinical characteristics of these nine newborns are summarized in Table 2. In six of these nine newborns, CHD had been diagnosed by fetal ultrasound; on the initial echocardiogram screening performed by a pediatric cardiologist immediately after birth, two newborns were found to have TOF, one had PA, one had single ventricle, one had CoA, and one had atrial ventricular septal defect. The remaining three newborns (one with TAPVC and two with TOF) screened negative for CHD on fetal ultrasound but exhibited heart murmur and/or cyanosis at 12–24 h after birth, though none had cardiovascular collapse.

**Table 2:** Clinical characteristics of critical and serious congenital heart disease

CHD	Screening method	Clinical findings	Age at intervention		Outcome
			Cardiac catheterization	Surgery	
Critical CHD					
SV	FUS	Cyanosis (SpO_2_: 30%)	-	4 days	Alive
SV & CoA	FUS	No	-	15 days	Alive
Serious CHD					
TAPVC	PE	Heart murmur Cyanosis (SpO_2_: 91–94%)	-	29 days	Alive
PA & DORV	FUS	Heart murmur	-	1 month	Alive
AVSD	FUS	No	2 months	3 months	Alive
TOF	PE	Heart murmur	-	6 months	Alive
TOF	FUS	Heart murmur	4 months	10 months	Alive
TOF	PE	Heart murmur	6 months	10 months	Alive
TOF	FUS	No	7 months	11 months	Alive

*CHD* congenital heart disease, *FUS* fetal ultrasound, *PE* physical examination, *SV* single ventricle, *CoA* coarctation of aorta, *TAPVC* total anomalous pulmonary venous connection, *PA* pulmonary atresia, *DORV* double-outlet right ventricle, *AVSD* atrial ventricular septal defect, *TOF* tetralogy of Fallot, *SpO*_*2*_ oxygen saturation

## Discussion

### Efficacy of postnatal echocardiographic screening for CCHD

In this study, postnatal echocardiographic screening did not help improve the detection rate of critical or serious CHD in newborns without prenatal diagnosis or clinical signs. Moreover, such early echocardiographic screening detected non-significant CHDs, which were not associated with clinical manifestations and which resolved spontaneously within the first 6 months of life.

Fetal ultrasound screening allows for early detection of CHD [12–14], especially when conducted routinely in tertiary care centers [15–17]. In our hospital, fetal ultrasound screening is routinely conducted for all pregnant women. When fetal CCHD is recognized by an obstetrician, the pregnant woman is referred for detailed fetal assessment by a pediatric cardiologist. Previous studies have also suggested that collaboration with a pediatric cardiologist may improve the sonographic detection rate of fetal CHD [18, 19]. In this setting, postnatal echocardiographic screening may not significantly improve the early detection rate of CCHD. However, the detection rate of CHD by prenatal ultrasound can be limited by fetal gestational age or position, maternal obesity, and operator experience [3, 16, 20]. In fact, of the newborns delivered during the study period, all except six screened negative for CHD on prenatal ultrasound, while three were postnatally diagnosed as having critical or serious CHD despite having screened negative on prenatal ultrasound; these three newborns had clinical signs indicative of CHD postnatally, including heart murmur and/or cyanosis. However, in newborns with CCHD, murmurs do not always manifest prior to hospital discharge, due to specific anatomic features, elevated pulmonary vascular resistance, or reduced contractility [21, 22]. Cyanosis may also be difficult to detect in newborns with mild desaturation or with dark or anemic skin color, as well as when lighting in the room is poor [8, 23]. Because of these problems, routine neonatal examination fails to detect CHD in about half of cases [24, 25].

Although postnatal echocardiography is the gold standard for diagnosing CHD, it has serious limitations when used as a screening tool, as we demonstrated in the present study. Moreover, while echocardiographic screening could be implemented in our hospital relatively easily (around 600 newborns delivered every year), implementing such a screening strategy is not feasible in large-volume facilities, as it would lead to an unacceptable increase in workload for pediatric cardiologists. In addition, employing experienced pediatric cardiologists to perform screening echocardiography for all newborns and to follow up all test positives would incur prohibitive costs. Upon evaluating the effectiveness and costs of several current strategies for CCHD screening of newborns, Knowles et al. found that echocardiographic screening was the most costly strategy, concluding that such a screening strategy is unlikely to be cost-effective [26, 27]. In addition, our present results confirm the fact that this screening strategy has a high rate of false-positives, which may induce adverse psychological effects in parents. To minimize the rate of false-positives, echocardiographic screening could be conducted later (e.g., at a few months from birth); however, some newborns with unsuspected CCHD may experience sudden manifestations such as cardiovascular collapse or death within 2 months of birth, and would thus not have the opportunity to be screened for CCHD.

### Strengths and limitations of the study

One important strength of the present study is that echocardiographic screening was conducted by experienced pediatric cardiologists, who evaluated thousands of neonates both before and during the study period. Another strength is that the study included almost 3500 newborns. On the other hand, our study has several potential limitations. First, similar studies regarding postnatal echocardiographic screening were performed previously [9–11]. Such studies investigated the prevalence of CHD and the efficacy of screening for CHD, but not the efficacy of screening for CCHD. Moreover, these previous studies only analyzed data pertaining to short follow-up periods, which makes it difficult to assess the prognosis of most patients. In our study, the follow-up period of patients with clinically significant CHD was at least 2 years. Among these previous studies, one was conducted at our institution and covered the period between 2005 and 2010, reporting a high prevalence of CHD in an unselected series of consecutive newborns, including those with significant prematurity, chromosomal or genetic abnormality, prenatally diagnosed CHD, and/or clinical signs or symptoms of CHD [10]. On the contrary, in the present study, we evaluated the efficacy of postnatal echocardiography for detecting unsuspected CCHD in a well-baby population and found a low incidence of CHD. Another potential limitation is that our study was conducted at a single tertiary hospital, and thus selection bias could not be excluded. In fact, the incidence of CCHD in this population was lower than previously reported [2, 3]. Finally, we did not have data regarding the outcomes of newborns who screened negative on postnatal echocardiography and physical examination.

### Alternative screening strategy for CCHD

Current evidence supports the introduction of pulse oximetry screening for CCHD in asymptomatic newborns before hospital discharge [6, 7], suggesting that pulse oximetry should be used as a non-invasive, inexpensive, and less time-consuming screening tool [5, 6]. However, there are technical limitations associated with oximetry measurements in newborns, resulting in a high rate of false-negatives; specifically, approximately a quarter of newborns with CCHD will not be identified by this method. The lesions most commonly missed on pulse oximetry evaluations are those causing obstruction of the aortic outflow (e.g., CoA and IAA), which may not necessarily be associated with hypoxemia [3, 8]. Such lesions are also frequently missed on prenatal ultrasound and routine physical examination [28]. Thus, in newborns with such lesions, echocardiographic screening may be the only tool suitable for early diagnosis.

## Conclusions

In our tertiary care hospital, echocardiographic screening did not help improve the detection rate of CCHD in newborns without prenatal diagnosis or clinical signs of CHD, and was moreover associated with a high rate of false-positives. In light of these findings, as well as considering the high cost and increase in workload for pediatric cardiologists, we conclude that echocardiography might not represent a feasible routine screening strategy for CCHD. Instead, it may be useful to employ pulse oximetry in conjunction with fetal ultrasound and postnatal physical examination. Our institution is planning to introduce such a screening strategy, although further research is warranted in order to inform policy.

## Abbreviations

AR: Aortic regurgitation
ASD: Atrial septal defect
AVSD: Atrial ventricular septal defect
CCHD: Critical congenital heart disease
CHD: Congenital heart disease
CoA: Coarctation of the aorta
DORV: Double-outlet right ventricle
IAA: Interruption of the aortic arch
MR: Mitral regurgitation
NICU: Neonatal intensive care unit
PA: Pulmonary atresia
PDA: Patent ductus arteriosus
PS: Pulmonary stenosis
SV: Single ventricle
TAPVC: Total anomalous pulmonary venous connection
TOF: Tetralogy of Fallot
TR: Tricuspid regurgitation
VSD: Ventricular septal defect
